# The Relationship between Locomotive Syndrome and Depression in Community-Dwelling Elderly People

**DOI:** 10.1155/2017/4104802

**Published:** 2017-04-05

**Authors:** Misa Nakamura, Hiroshi Hashizume, Sachiko Nomura, Ryohei Kono, Hirotoshi Utsunomiya

**Affiliations:** ^1^Department of Rehabilitation, Osaka Kawasaki Rehabilitation University, 158 Mizuma, Kaizuka, Osaka 597-0104, Japan; ^2^Department of Orthopedic Surgery, Wakayama Medical University, 811 Kimiidera, Wakayama 641-8510, Japan; ^3^Department of Strategic Surveillance for Functional Food and Comprehensive Traditional Medicine, Wakayama Medical University, 811 Kimiidera, Wakayama, Wakayama 641-8510, Japan

## Abstract

Locomotive syndrome (LS) is a concept that refers to the condition of people requiring healthcare services because of problems associated with locomotion. Depression is a major psychiatric disease among the elderly, in addition to dementia. The purpose of this study was to determine the association between LS and depression. The study participants were 224 healthy elderly volunteers living in a rural area in Japan. LS was defined as scores ≥ 16 on the 25-question Geriatric Locomotive Function Scale (GLFS-25). Depression was defined as scores ≥ 5 on the 15-item Geriatric Depression Scale (GDS-15). Height and body weight were measured. The prevalence of LS and depression was 13.9% and 24.2%, respectively. Compared with the non-LS group, the LS group was older, was shorter, had a higher BMI, and had higher GDS-15 scores. Logistic regression analysis showed that participants with GDS-15 scores ≥ 6 had higher odds for LS than those with GDS-15 scores < 6 (odds ratio [OR] = 4.22). Conversely, the depression group had higher GLFS-25 scores than the nondepression group. Participants with GLFS-25 scores ≥ 5 had higher odds for depression than those with GLFS-25 scores < 5 (OR = 4.53). These findings suggest that there is a close relationship between LS and depression.

## 1. Introduction

In our worldwide aging society, it is important that elderly people maintain physical and mental function to avoid health problems. Depression late in life is one of the most common mental disorders in old age [1]. According to research on community-dwelling older adults, the proportion of individuals reporting depressive symptoms ranges from 2.8% to 35% [2]. Depression in the elderly is a serious risk factor for becoming bedridden and requiring long-term care and is closely related to falls, as well as both quality of life (QOL) and activities of daily living (ADL) [3, 4]. In fact, it is reported that the death rate of people with depression is 1.8 times that of nondepressed people [5]. There is therefore an urgent need for medicine and social policy to find ways of reducing and preventing depression in older adults in the community.

Motor function decreases with aging, which also leads to a decrease in ADL and QOL. There is a close relationship between the physical activity level of elderly living in the community and their health-related QOL [6, 7].

Previous studies on depression and physical function of the elderly have reported that mental health is better with higher physical activity [8]. Furthermore, the higher the level of physical activity in middle age, the lower the degree of depression in older age [9, 10]. Many studies have also reported that exercise is efficacious in reducing depressive symptoms in older people [11].

A concept referred to as locomotive syndrome (LS) was proposed by the Japanese Orthopedic Association in order to help identify middle-aged and older adults who may be at high risk of requiring healthcare services because of problems associated with locomotion. Some causes of LS are reduced muscle strength and balance associated with aging and locomotive pathologies including osteoporosis, osteoarthritis, and sarcopenia [12]. The proportion of the Japanese population with LS (47 million) is estimated to be more than twice that with metabolic syndrome (20 million) [13, 14].

There are few reports on the relationship between depression and LS in the elderly. Therefore, the purpose of this study was to determine the association between LS and depression with the goal of helping prevent LS and depression in community-dwelling elderly.

## 2. Methods

### 2.1. Participants

This study was conducted in a rural area (Tanabe City, Wakayama Prefecture, Japan) between January 2013 and March 2015. The study inclusion criteria were as follows: (1) Japanese, aged ≥ 60 years, (2) able to walk independently, and (3) living at home and being capable of self-care in own home. All participants initially underwent measurement of body composition, namely, height (m) and body weight (kg), and then evaluation of LS status using a self-administered questionnaire, the 25-item Geriatric Locomotive Function Scale (GLFS-25) score at a public hall where a “lecture meeting and checkup for health,” supported by the local government in Tanabe, Wakayama, was held. Afterwards, 259 (82 men and 177 women) participants were asked to complete a self-administered questionnaire of the 15-item Geriatric Depression Scale (GDS-15) at home and return it by mail. A stamped envelope was provided to encourage return of the questionnaire. A total of 244 participants (mean age 68.3 ± 6.4 years; range 60–90 years) returned the questionnaire. All participants provided written informed consent to use their data in the study. This study was performed in accordance with the Declaration of Helsinki and was approved by the Ethics Committee of Wakayama Medical University (reference number 1005).

### 2.2. Measurement of Variables

Body weight was measured using a body composition scale (KaradaScan362, OMRON Co., Kyoto, Japan) while participants were wearing normal indoor clothing without shoes. BMI (kg/m^2^) was calculated from height and body weight.

### 2.3. Assessment of LS Status

LS status was evaluated using GLFS-25 score. The GLFS-25 is a self-administered questionnaire that consists of four questions on pain during the last month, 16 questions on ADL during the last month, three questions on social function during the last month, and two questions on mental health status during the last month [15]. All 25 items are scored from 0 (no impairment) to 4 (severe impairment), and the total score ranges from 0 to 100. The cutoff score for LS is 16 points; therefore, scores of ≥16 are evaluated as LS [15].

### 2.4. Evaluation of Depression

Depression status was assessed using GDS-15, which is a commonly used instrument for depression screening in the general geriatric population. The GDS-15 is a yes/no questionnaire that does not focus on somatic symptoms and does not contain any questions about suicide. All 15 items are scored as either 0 or 1, and the total score ranges from 0 to 15. Scores from 5 to 9 indicate minor depressive disorder and scores ≥ 10 indicate major depressive disorder [16]. The cutoff score for depression is 5 points; therefore, scores of ≥5 are evaluated as depression.

### 2.5. Statistical Analysis

Participants were categorized into a depression group (GDS-15 score ≥ 5) or a nondepression group (GDS-15 score < 5) and into an LS group (GLFS-25 score ≥ 16) or a non-LS group (GLFS-25 score < 16). The independent variables were compared between groups. For numerical variables (age, height, body weight, BMI, GLFS-25 score, and GDS-15 score), the normality of distribution and homogeneity of variance were tested prior to comparison across groups. Student's* t*-test was used when the assumptions of normal distribution and homogeneity of variance were met in both groups, Welch's* t*-test was used when the assumption of normal distribution was met but the assumption of homogeneity of variance was not, and the Wilcoxon signed-rank test was used when the data were not normally distributed. Pearson's chi-square test was used to compare the number of males and females in the LS and depression groups, the number of participants with depression in the LS and non-LS groups, and the number of participants with LS in the depression and nondepression groups. The GLFS-25 threshold score for discriminating the depression group and the nondepression group and the GDS-15 threshold score for discriminating the LS group and the non-LS group were evaluated using receiver-operating curve (ROC) analysis. The odds ratio (OR) of depression for the GLFS-25 threshold score was calculated using multiple logistic regression analysis involving age, sex, and BMI, and the OR of LS for the GDS-15 threshold score was calculated using the chi-square test. Statistical analysis was conducted using JMP 11 (SAS Institute, Cary, NC). All statistical tests were two-tailed, and a significance level of 0.05 was used.

## 3. Results

Thirty-four participants (13.9%) had a GLFS-25 score ≥ 16 and were classified as having LS, while 67 (27.5%) had a GDS-15 score ≥ 5 and were classified as having depression (Table 1).

Compared with the non-LS group, the LS group was older, was shorter, had a higher BMI, and had higher GDS-15 scores (Table 2). ROC analysis of GDS-15 scores identified a threshold score of 6 for discriminating the LS and non-LS groups (area under the curve (AUC) = 0.65, sensitivity = 41.18%, and specificity = 72.63%). Logistic regression analysis showed that participants with GDS-15 scores ≥ 6 had higher odds for LS than those with GDS-15 scores < 6 (OR = 4.22, 95% confidence interval [CI] = 1.80–9.91; *p* < 0.001) (Table 3).

The depression group had higher GLFS-25 scores than the nondepression group (Table 4). ROC analysis of GLFS-25 scores showed a threshold score of 5 for discriminating the depression and nondepression groups (AUC = 0.68, sensitivity = 82.09%, and specificity = 68.19%). Logistic regression analysis showed that participants with a GLFS-25 score ≥ 5 had higher odds for depression than those with a GLFS-25 score < 5 (OR = 4.53, 95% CI = 2.34–9.41; *p* < 0.0001) (Table 5).

## 4. Discussion

Our results showed that participants with LS had a shorter height, higher BMI, and higher prevalence of depression than participants without LS. Participants with depression had a higher prevalence of LS than participants without depression. The finding that LS is related to age, height, and BMI is consistent with our previous study of elderly female subjects [17].

Recently, Ikemoto et al. [18] reported that comparative analysis between LS and non-LS subjects revealed significant differences in the degree of depression with age and physical function. In our study, it was suggested that the relationship between LS and depression is bidirectional and that the threshold score for LS using the GDS-15 was 5 and the threshold score for depression using the GLFS-25 score was 6. Furthermore, the ORs of participants with scores above these thresholds were 4.22 for LS and 4.53 for depression, respectively.

The amount of sleep, presence of a chronic disease, annual income, education level, weekly consumption of meat and beans/bean products, BMI, and level of physical activity were significantly associated with depression symptoms in previous studies [19, 20]. There are many reports on physical capacity and depression, and in particular, it has been shown that grip strength, lower limb muscular strength, balance ability, and walking ability are related to depression [21–23]. Based on this, it is conceivable that a decline in physical function represents a risk factor for depression. On the other hand, Roshanaei-Moghaddam et al. [24] reviewed the results of longitudinal studies in the literature and found that depression may be a significant risk factor for decreased level of physical exercise.

The concept of LS was proposed by the Japanese Orthopedic Association in 2007 to identify individuals at high risk of requiring nursing care due to problems with their locomotive system [12]. Many reports have suggested that the GLFS-25 score strongly correlates with several measures of physical performance, including the unipedal stance test and the timed up and go test [23, 25–28].

There is a relationship between depression and locomotive disorder, osteoporosis, lumbar spinal stenosis, and fracture [29–31]. Endocrine factors such as depression-induced hypersecretion of corticotropin-releasing hormone and hypercortisolism, hypogonadism, growth hormone deficiency, and increased concentration of circulating interleukin 6 might play a crucial role in the bone loss observed in subjects suffering from major depression [29]. Previous research found that preoperative depression is likely a prognostic factor for postoperative lumbar spinal stenosis-related symptom severity and disability at various follow-up points [30].

A recent study reported that pain status associated with lumbar spinal stenosis and the knee joint affected GLFS-25 scores [32]. Among individuals with arthritis, depression is associated with increased pain, work disability, and functional decline [33, 34]. Similarly, pain can also lead to depression, suggesting that the relationship between depression and pain is bidirectional [35].

**Figure 1:**
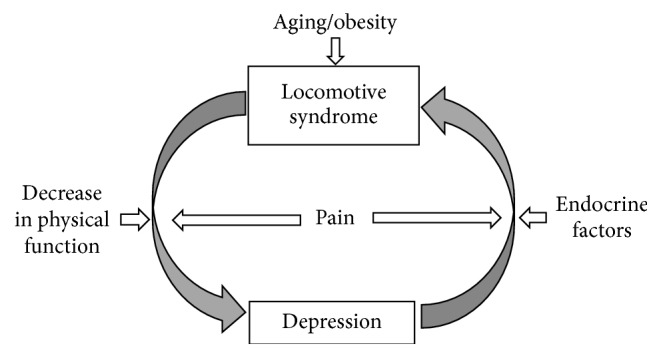
Association between locomotive syndrome and depression.

From the above, it is thought that a chainwise vicious cycle such as LS causes depression due to pain or decreased physical function, and furthermore, depression causes LS due to pain or hormonal change (Figure 1). Therefore, management strategies for depression and LS should take into account both depressive symptoms and LS to improve health-related QOL.

This study has several limitations that should be taken into consideration. First, it cannot be concluded that LS and depression have a bidirectional relationship from the results of the present cross-sectional study alone. However, it is rationally presumed that the two influence each other based on the literature. The present results showed the ORs of GDS-15 and GLFS-25 scores by analyzing objective variables with and without the presence of both LS and depression. Therefore, we believe that this study contributes new information about both the mental risk seen from the perspective of locomotion and the risk to locomotion from the perspective of mental health. In the future, longitudinal studies of LS and depression with intervention and follow-up are required. Secondly, the sample size of 244 was small; this number only represents about 1.0% of all people aged between 60 between 90 years in Tanabe City. Third, because of the sampling bias, we cannot assume the participants of this study represent the general elderly population in Japan. Fourth, since we have no information on participants' low back pain, knee pain, and physical inactivity as confounding factors for LS, or for nutritional balance, family relationships, economic situation, amount of physical activity, and cognitive function as confounding factors for depression, we cannot conclude how these factors may have affected the results.

## 5. Conclusion

It is clear that there is a relationship between LS and depression, and the thresholds for LS and depression are a GDS-15 score of 5 and a GLFS-25 score of 6, respectively. Furthermore, participants with scores above these thresholds had ORs of 4.22 for LS and 4.53 for depression, respectively. Therefore, management strategies for LS and depression should take into accounts both depressive symptoms and LS to improve health-related QOL among community-dwelling elderly.

## Figures and Tables

**Table 1:** Characteristics of the study participants.

Variables for components	* *Overall
Participants	* *244
Male (%)	* *79 (32.38)
Age (years; mean (SD^a^))	* *68.30 (6.41)
Height (cm; mean (SD))	* *155.17 (12.30)
Body weight (kg; mean (SD))	* *57.42 (10.89)
Body mass index (kg/m^2^; mean (SD))	* *23.64 (3.33)
GLFS-25^b^ (points; mean (SD))	* *8.65 (9.18)
LS^c^ (%)	* *34 (13.9)
GDS-15^d^ (points; mean (SD))	* *3.09 (2.91)
Depression (%)	* *67 (27.46)

^a^SD: standard deviation

^b^GLFS-25: Geriatric Locomotive Function Scale

^c^LS: locomotive syndrome

^d^GDS-15: Geriatric Depression Scale.

**Table 2:** Comparison of characteristics between the non-LS^a^ and LS^b^ groups.

	Non-LS	LS	*p* value
Participants (%)	210 (86.07)	34 (13.93)	
Male (%)	73 (34.76)	6 (17.65)	0.0479^d^
Age (years; mean (SD^c^))	67.72 (6.24)	71.91 (6.37)	0.0005^e^
Height (cm; mean (SD))	156.49 (8.15)	147.06 (24.79)	0.0002^e^
Body weight (kg; mean (SD))	57.53 (10.86)	56.74 (11.20)	0.6952^f^
Body mass index (kg/m^2^; mean (SD))	23.42 (3.20)	24.88 (3.84)	0.0246^e^
GDS-15 (points; mean (SD))	2.85 (2.72)	4.62 (3.53)	0.0005^e^
Depression	52 (24.76)	15 (44.12)	0.0190^d^

^a^Non-LS: non-locomotive syndrome, GLFS-25 score < 16 points

^b^LS: locomotive syndrome, GLFS-25 score ≥ 16 points

^c^SD: standard deviation

^d^Pearson's chi-square test

^e^Wilcoxon signed-rank test

^f^Student's *t*-test.

**Table 3:** Evaluation of odds ratio for LS^a^ according to GDS-15^b^ score.

	Above or below the threshold value	Odds ratio (95% CI^c^)	*p* value
GDS-15 (points)	<6	1	0.0004
	≥6	4.22 (1.80–9.91)	

Multiple logistic regression analysis involving age, sex, and BMI was performed.

^a^LS: locomotive syndrome, GLFS-25 score

^b^GDS-15: Geriatric Depression Scale

^c^CI: confidence interval.

**Table 4:** Comparison of characteristics between the nondepression and depression groups.

	Nondepression	Depression	*p* value
Participants (%)	177 (72.5)	67 (27.5)	
Male (%)	66 (35.68)	13 (22.03)	0.8319^d^
Age (years; mean (SD^a^))	68.11 (6.43)	68.82 (6.40)	0.3937^e^
Height (cm; mean (SD))	154.85 (13.57)	156.04 (8.08)	0.6562^e^
Body weight (kg; mean (SD))	57.43 (11.22)	57.40 (10.03)	0.9837^f^
Body mass index (kg/m^2^; mean (SD))	23.63 (3.38)	23.59 (3.21)	0.9360^e^
GLFS-25^b^ (points; mean (SD))	7.28 (7.81)	12.27 (11.36)	<0.0001^e^
LS^c^	19 (10.27)	15 (25.42)	0.0190^d^

^a^SD: standard deviation

^b^GLFS-25: Geriatric Locomotive Function Scale

^c^LS: locomotive syndrome

^d^Pearson's chi-square test

^e^Wilcoxon signed-rank test

^f^Student's *t*-test.

**Table 5:** Evaluation of odds ratio for depression according to GLFS-25^a^ score.

	Above or below the threshold value	Odds ratio (95% CI^b^)	*p *value
GLFS-25 (points)	<5	1	<0.0001
	≥5	4.53 (2.34–9.41)	

The chi-square test was performed.

^a^GLFS-25: Geriatric Locomotive Function Scale

^b^CI: confidence interval.

## References

[1] Tsuruoka Y., Takahashi M., Suzuki M., Sato K., Shirayama Y. (2016). Utility of the Neurobehavioral Cognitive Status Examination (COGNISTAT) in differentiating between depressive states in late-life depression and late-onset Alzheimer’s disease: a preliminary study. *Annals of General Psychiatry*.

[2] Beekman A. T. F., Copeland J. R. M., Prince M. J. (1999). Review of community prevalence of depression in later life. *British Journal of Psychiatry*.

[3] Wada T., Ishine M., Sakagami T. (2005). Depression, activities of daily living, and quality of life of community-dwelling elderly in three Asian countries: Indonesia, Vietnam, and Japan. *Archives of Gerontology and Geriatrics*.

[4] Wada T., Ishine M., Sakagami T. (2004). Depression in Japanese community-dwelling elderly—prevalence and association with ADL and QOL. *Archives of Gerontology and Geriatrics*.

[5] Cuijpers P., Schoevers R. A. (2004). Increased mortality in depressive disorders: a review. *Current Psychiatry Reports*.

[6] Dunn A. L., Dishman R. K. (1991). Exercise and the neurobiology of depression. *Exercise and Sport Sciences Reviews*.

[7] Halaweh H., Willen C., Grimby-Ekman A., Svantesson U. (2015). Physical activity and health-related quality of life among community dwelling elderly. *Journal of Clinical Medicine Research*.

[8] Strawbridge W. J., Deleger S., Roberts R. E., Kaplan G. A. (2002). Physical activity reduces the risk of subsequent depression for older adults. *American Journal of Epidemiology*.

[9] Chang M., Snaedal J., Einarsson B. (2016). The association between midlife physical activity and depressive symptoms in late life: age gene/environment susceptibility-Reykjavik Study. *Journals of Gerontology-Series A Biological Sciences and Medical Sciences*.

[10] Dugan S. A., Bromberger J. T., Segawa E., Avery E., Sternfeld B. (2015). Association between physical activity and depressive symptoms: midlife women in SWAN. *Medicine and Science in Sports and Exercise*.

[11] Catalan-Matamoros D., Gomez-Conesa A., Stubbs B., Vancampfort D. (2016). Exercise improves depressive symptoms in older adults: an umbrella review of systematic reviews and meta-analyses. *Psychiatry Research*.

[12] Nakamura K. (2008). A “super-aged” society and the “locomotive syndrome”. *Journal of Orthopaedic Science*.

[13] Ministry of Health Labour and Welfare http://www.mhlw.go.jp/english/wp/wp-hw2/part2/p3_0024.pdf.

[14] Yoshimura N., Muraki S., Oka H. (2009). Prevalence of knee osteoarthritis, lumbar spondylosis, and osteoporosis in Japanese men and women: the research on osteoarthritis/osteoporosis against disability study. *Journal of Bone and Mineral Metabolism*.

[15] Seichi A., Hoshino Y., Doi T., Akai M., Tobimatsu Y., Iwaya T. (2012). Development of a screening tool for risk of locomotive syndrome in the elderly: the 25-question geriatric locomotive function scale. *Journal of Orthopaedic Science*.

[16] Shiekh J., Yesavage J., Brink T. (1986). Clinical gerontologyv: a gutide to assessnment andintervention. *Geriatric Depression Scale; Recent Findings and Development of a Short Version*.

[17] Nakamura M., Kobashi Y., Hashizume H. (2016). Locomotive syndrome is associated with body composition and cardiometabolic disorders in elderly Japanese women. *BMC Geriatrics*.

[18] Ikemoto T., Inoue M., Nakata M. (2016). Locomotive syndrome is associated not only with physical capacity but also degree of depression. *Journal of Orthopaedic Science*.

[19] Tanaka H., Sasazawa Y., Suzuki S., Nakazawa M., Koyama H. (2011). Health status and lifestyle factors as predictors of depression in middle-aged and elderly Japanese adults: a seven-year follow-up of the Komo-Ise cohort study. *BMC Psychiatry*.

[20] Zhou X., Bi B., Zheng L. (2014). The prevalence and risk factors for depression symptoms in a rural Chinese sample population. *PLoS ONE*.

[21] Brown W. J., Ford J. H., Burton N. W., Marshall A. L., Dobson A. J. (2005). Prospective study of physical activity and depressive symptoms in middle-aged women. *American Journal of Preventive Medicine*.

[22] Demakakos P., Cooper R., Hamer M., de Oliveira C., Hardy R., Breeze E. (2013). The bidirectional association between depressive symptoms and gait speed: evidence from the English Longitudinal Study of Ageing (ELSA). *PLoS ONE*.

[23] Fukumori N., Yamamoto Y., Takegami M. (2015). Association between hand-grip strength and depressive symptoms: Locomotive Syndrome and Health Outcomes in Aizu Cohort Study (LOHAS). *Age and Ageing*.

[24] Roshanaei-Moghaddam B., Katon W. J., Russo J. (2009). The longitudinal effects of depression on physical activity. *General Hospital Psychiatry*.

[25] Muramoto A., Imagama S., Ito Z., Hirano K., Ishiguro N., Hasegawa Y. (2012). Physical performance tests are useful for evaluating and monitoring the severity of locomotive syndrome. *Journal of Orthopaedic Science*.

[26] Seichi A., Hoshino Y., Doi T. (2014). Determination of the optimal cutoff time to use when screening elderly people for locomotive syndrome using the one-leg standing test (with eyes open). *Journal of Orthopaedic Science*.

[27] Yoshimura N., Oka H., Muraki S. (2011). Reference values for hand grip strength, muscle mass, walking time, and one-leg standing time as indices for locomotive syndrome and associated disability: the second survey of the ROAD study. *Journal of Orthopaedic Science*.

[28] Nakamura M., Hashizume H., Oka H. (2015). Physical performance measures associated with locomotive syndrome in middle-aged and older Japanese women. *Journal of Geriatric Physical Therapy*.

[29] Cizza G., Ravn P., Chrousos G. P., Gold P. W. (2001). Depression: a major, unrecognized risk factor for osteoporosis?. *Trends in Endocrinology & Metabolism*.

[30] McKillop A. B., Carroll L. J., Battié M. C. (2014). Depression as a prognostic factor of lumbar spinal stenosis: a systematic review. *Spine Journal*.

[31] Zong Y., Tang Y., Xue Y. (2016). Depression is associated with increased incidence of osteoporotic thoracolumbar fracture in postmenopausal women: a prospective study. *European Spine Journal*.

[32] Chiba D., Tsuda E., Wada K. (2016). Lumbar spondylosis, lumbar spinal stenosis, knee pain, back muscle strength are associated with the locomotive syndrome: rural population study in Japan. *Journal of Orthopaedic Science*.

[33] Dickens C., Creed F. (2001). The burden of depression in patients with rheumatoid arthritis. *Rheumatology*.

[34] Dunlop D. D., Semanik P., Song J., Manheim L. M., Shih V., Chang R. W. (2005). Risk factors for functional decline in older adults with arthritis. *Arthritis and Rheumatism*.

[35] Kroenke K., Wu J., Bair M. J., Krebs E. E., Damush T. M., Tu W. (2011). Reciprocal relationship between pain and depression: a 12-month longitudinal analysis in primary care. *Journal of Pain*.

